# Braque and Kokoschka: Brain Tissue Injury and Preservation of Artistic Skill

**DOI:** 10.3390/bs7030056

**Published:** 2017-08-19

**Authors:** D. W. Zaidel

**Affiliations:** Department of Psychology, University of California at Los Angeles, 405 Hilgard Avenue, Los Angeles, CA 90095, USA; dahliaz@g.ucla.edu

**Keywords:** brain and art, neuropsychology and art, brain damage, artists

## Abstract

The neural underpinning of art creation can be gleaned following brain injury in professional artists. Any alteration to their artistic productivity, creativity, skills, talent, and genre can help understand the neural underpinning of art expression. Here, two world-renown and influential artists who sustained brain injury in World War I are the focus, namely the French artist Georges Braque and the Austrian artist Oskar Kokoschka. Braque is particularly associated with Cubism, and Kokoschka with Expressionism. Before enlisting, they were already well-known and highly regarded. Both were wounded in the battlefield where they lost consciousness and treated in European hospitals. Braque’s injury was in the left hemisphere while Kokoschka’s was in the right hemisphere. After the injury, Braque did not paint again for nearly a whole year while Kokoschka commenced his artistic works when still undergoing hospital treatment. Their post-injury art retained the same genre as their pre-injury period, and their artistic skills, talent, creativity, and productivity remained unchanged. The quality of their post-injury artworks remained highly regarded and influential. These neurological cases suggest widely distributed and diffuse neural control by the brain in the creation of art.

## 1. Background

The recent decade has seen a remarkable expansion in the understanding of the neural underpinning of the arts. This can largely be attributed to a major conceptual breakthrough in which the aesthetic reaction of viewers (aesthetic experience) to art was determined to be separate from the art creation/production process. The techniques for measuring these two aspects of art apply different scientific strategies. Functional neuroimaging of healthy viewers reacting to artwork provides inroads into the neural underpinning of aesthetic evaluation [1,2,3,4,5]. The general consensus from these studies is that multiple brain regions and pathways are involved in aesthetic evaluation. This suggests that layers of our concepts of art aesthetics need to be peeled in order to understand how each active brain region contributes to the aesthetic experience. In this special issue, “Neuropsychology of Art,” three papers emphasize the viewers’ aesthetic reactions through the examination of neuroimaging empirical data [6], the examination of neuroimaging literature [7], and the examination of the reactions in light of mirror neurons and psychoanalytic considerations [8].

For the creation of the art itself, observations of established artists with acquired brain injury provide the insights necessary to explore the nature of the neural underpinning of the production aspect. The questions pursued concern whether or not single or multiple neural circuitries are involved, the hemispheric specialization role [9], as well as any post-injury alterations in the artist regarding skill, talent, personal oeuvre, and creativity [10,11,12]. The focus in the present paper is on the creation aspect of art through a discussion of world-renown artists, Braque and Kokoschka, both of whom sustained penetrating head injuries in World War I (WWI).

Inferring cognitive functions of the mind from patients with brain lesions has been a widely accepted method in brain research. The same strategy applies to art. An early assumption, not founded on empirical studies, was that the right hemisphere specializes in producing and aesthetically reacting to art. Unfortunately, the assumption became prevalent, and eventually was challenged [13]. Indeed, close examination of the artworks of artists with brain injury revealed otherwise [10,14,15,16,17]. What was unraveled from such artists suggests that unilateral injury in the right hemisphere does not abolish the ability to produce art, something one would expect if the right hemisphere specialized in this function, and that damage in either hemisphere does not lead to deficits in the artistic ability, talent, skill, or creativity of established professional artists [10]. The current emerging view is that artworks tap functions specialized in both hemispheres and involve widely distributed neural pathways that contribute to its production.

The effects of brain injury on artists continue to fascinate and provide insights. In the cases described below, the main interests lie in any alteration of their pre-injury genre, their artistic skills, and their creativity. The neurological cases of Braque and Kokoschka have not been discussed in detail previously. They are important because both were highly accomplished and influential artists, both before and after their brain injury.

## 2. Georges Braque

Georges Braque (1882–1963) was an innovative, world-renown, and highly influential French artist linked mainly with the school of Cubism. He was a painter, draughtsman, sculptor, and engraver whose early interest in Cubism reflected the influence of artist Paul Cezanne [18]. “Braque found in Cezanne a fragmented plane, straddling nature and painting that was to become the principles governing his own oeuvre” [18] (p. 25). In his own Cubist art, Braque expressed the idea of object fragmentation combined with emphasis on geometrical forms. Through his personal and professional relationship with Pablo Picasso, for over six years Cubist characteristics were enriched and developed [19]. The volume of Cubist artworks produced by these two artists was prolific, innovative, and influential [20,21].

Then, in 1914, WWI broke out, and in August of that year Braque enlisted in the French army [22]. On 11 May 2015, in one of the battles in France, he sustained a head wound from a piece of shrapnel in a section of the battlefield that lay outside the trench. When he lost consciousness as a result of the shrapnel, he was thought to have died. Only a day later, a search party found and rescued him. The medical treatment in the hospital consisted of drilling a hole into his skull (a procedure known as trepanation), which resulted in Braque experiencing temporary blindness and a two-day coma from which he recovered only around 13 May [22]. His physical recovery lasted nearly a year, and during that time reportedly he did not paint.

A formal neurological report of his hospital treatment has not been published, but details of his medical and physical condition are known from descriptions offered by himself, several of his friends, and his wife [19,21,23,24]. Importantly, further critical clues can be deduced from a single photograph: At one point in 1915, when Braque was convalescing in Sorgues, a photograph of a seated Braque was taken at Henri Lauren’s studio in the same town [21]. We can see a rather thick absorbent gauze pad over a circumscribed area in the left side of his head, suggesting that it covered the wound. The gauze was held in place by a large bandage wrapped around his head. Assuming that the published photograph was not mirror-reversed, the wound was in the left side of the head. The gauzed region appears to correspond roughly to the left parietal lobe and possibly the superior temporal lobe. It does not appear to cover the left temple behind which Broca’s area lies, nor is it positioned so far back in the head to include the occipital lobe. Indeed, there has not been any mention by his friends and associates of aphasia or any other language disturbances, and similarly no reports of right hand or leg paralysis. Those are symptoms we would expect from damage to the left hemisphere. There has also been no mention of right spatial hemi-neglect, prosopagnosia, spatial agnosia, or left hand and leg paralysis, all symptoms we would expect if the right hemisphere were damaged. Whatever the localization of the damage in the left hemisphere, Braque did not paint for nearly a year afterwards.

Some comments on the medical practice of trepanation and head wounds in WWI are worthwhile. The practice then was applied to alleviate pressure caused by blood clots, to remove shell fragments, bullets, hair, abscesses, or to drain built-up liquid forming under the skull bone [25]. After burring, sometimes surgeons used their own fingers to remove foreign bodies, but if not done gently enough, this could cause injury to the brain tissue. Magnets were also used to remove metallic pieces. It is not known for certain how many holes were actually burred in his skull, but since with trepanation the goal was not to injure cerebral tissue, we should assume that no substantial tissue damage was inflicted in Braque’s case.

When he did resume painting, which was around the summer of 1917 [26], he picked up artistically where he left off at the start of the war, namely, his characteristic oeuvre of Cubism, and more particularly what is known as synthetic Cubism [18,22]. Not only were the geometric forms produced again, straight lines made with a ruler, objects overlapping each other, both animate and inanimate, but the pre-war technique of *papier collé* was applied as well [19]. “For although the picture space [in Cubism] had become independent of external reality, the subject matter had lost none of its rights. Quite the opposite—it had a greater function than ever, it was simply that the subject matter no longer had any meaning outside the picture. That was the fundamental principle that governed Braque’s work from 1914 until the end. The interruption caused by the First World War did not affect his development in any way, as has often been said” [18] (p. 124). Significantly for the present discussion, after the war he did not produce an entirely new artistic genre, nor did he return to his pre-Cubist genres (i.e., Fauvism, Neo-Impressionism). Indeed, he proceeded to develop and expand his artistic expression within the Cubist tradition for many years.

One can reasonably argue that his brain injury did not produce extensive tissue damage to have inflicted major deficits on his artistic cognition. With the absence of detailed medical reports of his case, the extent of the damage cannot be ascertained with certainty. However, from available non-medical behavioral descriptions made around the time of his injury, it is clear that he was unable to create art. This suggests that neural damage was present. Upon neural recovery, he returned to his art seemingly unscathed.

## 3. Oskar Kokoschka

Oskar Kokoschka (1886–1980) was a well-known Austrian artist who painted in the Expressionist tradition. He was also a poet and a playwright. In WWI, he fought on the side of Austria and suffered a head wound. On 29 August 1915, a bullet penetrated his skull during a Russian ambush on his troops in Sikiryczy, Ukraine. This wound, together with a bayonet lance into his lung, rendered him unconscious long enough to have been thought dead by others on his side of the battle. The bullet was not lodged in his brain, but had exited. In his autobiography [27], he describes the events that transpired during that battle: his left hand became paralyzed, presumably as a result of the head wound to the right hemisphere (he does not actually report the head side). Following his rescue, he reported that he had difficulties in walking and seeing. Both his balance and vision were affected by the head wound, and for the rest of his life he suffered from bouts of vertigo.

After a few months in the hospital, and despite all of his symptoms, he was considered fit to return to the battlefield, this time on Hungarian territory fighting against Italian troops. There, shells exploding near his position rendered him shell-shocked. Hungarian orderlies transported him to a hospital in Vienna where lesions in his cerebellum were inferred from X-rays. Normal gait balance is maintained by the cerebellum. To restore his balance, he underwent countless sessions of therapy consisting of induced painful spasms, which he found hard to endure to such an extent that thoughts of suicide formed in his mind [27]. In the fall of 1917, he was again subjected to painful experiments, this time in the Swedish laboratory of Nobel Prize winner Robert Barany. He reports that he stayed in Sweden for that purpose for a period of six to eight weeks, and during that time he continued to paint new works, still in the style of Expressionism.

To the best of my knowledge, the exact point of bullet penetration and its path of destruction in his brain have not been reported. The left hand paralysis he experienced on the battlefield was reversible. No mention of neuropsychological right hemisphere symptoms appears in the writings about him by others or in his own observations of himself. Whatever the nature of brain tissue damage caused by the bullet, it did not hamper his artistic talent and skills. Unlike Braque, who did not paint for a whole year following his injury, Kokoschka never ceased to paint and write, even while receiving medical treatments and convalescing from his wound. In hospitals in Vienna and Dresden he wrote plays and painted paintings, and similarly while in the hospital in Sweden he continued to paint. Important to the main argument here is that, despite damage to brain tissue, he continued with the same genre he had practiced prior to the war. He did not create new genres or new styles, and his motivation to create and innovate did not wane.

## 4. Other Established Artists

As with Braque and Kokoschka, numerous other professional prolific artists with damage to brain tissue continued to create art without loss of talent or skill [11]. One of these was a French artist P.A. from Marseilles. His case was described by Vigouroux et al. [28]. At the age of 66 years, he had a right hemisphere stroke that resulted in paralysis in the left side of his body as well as in a left hemi-neglect of space. The effects of the neglect were reflected in not painting in the spaces positioned to the left side of the canvas. Neurological patients with right hemisphere damage, especially if the parietal lobe is involved, commonly suffer from this condition; they attend only to the right half of their space, including their imaginary space formed in their mind’s eye [29,30]. There are classic examples of this phenomenon: Patients draw only the right half of a clock, flower, house, face, and other objects. They also fail to orient in the direction of a speaker who stands in their left side. In most such cases, the hemi-neglect is relatively short-lived, and this seems to have been the case with P.A.

Prior to the cerebral stroke, P.A. created paintings and drawings depicting scenery, everyday interactions of people, as well as nude women; some were painted with bright colors while some were in pale shades or as line drawings. In his post-injury artistic life, there was no alteration in these choices nor in his genre [28]. This case, too, shows that artistic talent and skill are not mediated by neural pathways specialized unilaterally in one hemisphere and that, instead, they are diffusely controlled by neural pathways in the brain.

## 5. General Discussion

Despite serious brain injury, the established and highly regarded visual artists described here resumed making their art without noticeable alterations in their personal oeuvre, talent, skill, or creativity—all elements that define the artistic endeavor. The genre represents the principle artistic choice of expression through the themes, concepts, ideas, design structure, organization, appearance, colors, and many more elements. The genre of choice communicates what is in the mind of the artist. At the cultural historical times of their work, their artworks continued to be highly regarded and influential. Braque, in particular, was a major figure in the art world at that time and his stature continued unabated. Previously, Boller reported preserved artistic abilities following a stroke in a French artist [15] and Vigouroux [28], reporting on artist P.A., remarked that his artistic interests and skills were not altered post-injury. Similarly, a review of many other post-injury artists revealed lack of alterations whether in the visual, musical, or literary arts, and regardless of etiology [10]. Importantly, the hemispheric laterality of the damage has not been found to cause detrimental effects on artistic abilities [11,13,31].

Ultimately, subjecting paintings and other visual artworks of artists with brain injury to microscopic computer digital analysis has the potential for revealing details in the artists’ pre- compared to post-injury methods. This has not yet been applied in the artists under discussion here. Future studies of digital analyses (e.g., [32,33]), might unravel additional interesting information.

Motoric hand use problems may arise as a result of the hemispheric side of the damage. With left hemisphere damage leading to right hand paralysis, right-handed artists switch to the use of the non-dominant hand (e.g., [31,34,35]). In such cases, the artistic cognition per se is not altered; the left hand lacks the same steady control and muscle strength exhibited pre-damage by the right hand, and the consequences of the switch to the non-dominant hand is reflected in brush pressure, stroke thickness, line curvature, or clay consistency (by sculptors), as well as in other features. Accommodation to the control of the hand itself is incorporated into the final appearance of the artwork. In such cases, the effect on the art, if any, is not central to the principle artistic cognition, which drives the artwork; peripheral motoric issues can be observed in manual activities in non-artists with similar brain injury as well. In any case, there was no switch of handedness as a result of the brain injury with Braque, Kokoschka, or P.A.

Similarly, brain injury can lead to specific perceptual deficits that are not unique to art or to artists. An example of this is hemi-neglect for the left half of space following right hemisphere injury. Visual artists with this type of injury typically do not paint in the left half of the canvas [11,14,16,31]. However, these cannot be considered to be art-related effects since the neglect symptoms are present in non-artists as well, and in many such cases the phenomenon is not permanent. Consequently, eventually, with the passage of post-injury time, artists fill in the left half of their canvas. Furthermore, artists’ production abilities appear unaffected by this type of brain injury. There is no loss of artistic talent and expressive abilities. This outcome suggests that the artistic cognitive endeavor, which includes the units listed above, can be controlled by undamaged regions and pathways and are likely to be neurally distributed in the brain.

In the great majority of cases, artists go on producing their works despite their brain injury [10]. What is it about the neural underpinning of the creation aspect of art that spares it from serious brain injury, unlike the brain localized effects on language, for example? A critical feature of the human brain is that it supports cognitive symbolic and abstract thinking as well as referential communicative abilities more than any other animal [36,37,38]. This is the underpinning of humans’ sophisticated language. Vocabulary and syntax together allow humans to make an infinite number of combinations that together convey a wide range of meaningful utterances. Furthermore, abstract and symbolic thinking is also the bases for art creation and appreciation. This can explain partly why only humans make art; it does not fully explain why brain injury largely spares the production of it. The answer may lie in what role art has come to play in human society. We need to take a broader perspective through the exploration of the evolutionary pressures on the human brain.

The evolutionary trajectory that led to the eventual emergence of *Homo sapiens* [39,40] involved overcoming survival hurdles in which social groups with emphasis on unity-for-survival played a major role [41,42]. Of all the hominins, *Homo sapiens* seem to be the only ones to make art [39]. For socially-oriented early human groups language became a highly efficient method of communication, and its evolution has deep roots going back to non-human primates tens of millions of years. Art, too, is a communicative system, but its roots cannot simply be traced to other primates; they do not produce art. Art supplements language by facilitating the sharing of emotions, experiences, ideas, thoughts, and symbols of social identity, and for these probable reasons it promoted bonding among the early group’s members in efforts to survive harsh environmental conditions with limited food sources. These could be some of the underlying reasons for how art became a form of communication that supplements language.

Unlike language, where neural control in the brain is highly localized (mainly in the left hemisphere), the advantage of art’s communicative format is that humans have increased their ability to express their inner and external life’s experiences through widely distributed systems in the brain. It explains in part why brain injury of the kind described here does not lead to significant alterations in art expression.
